# Implementing emergency department-based HIV testing in a low-resource setting: The value of a structured feasibility assessment tool

**DOI:** 10.4102/sajhivmed.v19i1.793

**Published:** 2018-07-16

**Authors:** Madeleine Whalen, Pamela Mda, Andy Parrish, Thomas C. Quinn, Richard Rothman, David Stead, Bhakti Hansoti

**Affiliations:** 1 Johns Hopkins Hospital, United States; 2 Department of Medicine, Faculty of Health Sciences, Walter Sisulu University, South Africa; 3 Department of Internal Medicine, Frere and Cecilia Makiwane Hospitals, South Africa; 4 Division of Intramural Research, National Institute of Allergy and Infectious Diseases, United States; 5 Division of Infectious Diseases, Johns Hopkins School of Medicine, Unite States; 6 Department of Emergency Medicine, Johns Hopkins University, United States

## Abstract

**Introduction:**

HIV is a worldwide health problem with continuing high rates of new infections in many parts of the world. This lack of progress in decreasing overall incidence rates has sparked innovative HIV testing strategies, including expansion of testing into the emergency department (ED) setting. Emergency departments have been shown to be high-yield testing venues in the United States and other developed world settings. The feasibility of expanding public health HIV services in the ED in limited-resource countries is unclear.

**Methods:**

We performed a cross-sectional feasibility assessment of a convenience sample of four hospitals in the Eastern Cape, South Africa. We administered three adapted interview tools from a previously field-tested survey instrument at each facility (total of 10 interviews) to gather an overview of the health facility, their HIV counselling and testing services, and their laboratory services.

**Results:**

All of the health facilities had access to basic commodities such as water and electricity. Many had severe human resource limitations and provided care to wide population catchment areas. In addition, there was little integration of HIV testing into current daily ED operations. Hospital staff identified numerous barriers to future ED testing efforts.

**Conclusions:**

Although control of the HIV epidemic requires innovative testing strategies and treatment, specific assessments are warranted on how to incorporate routine HIV testing into an acute care facility like the ED, which typically has many competing priorities. The use of a prospective structured tool incorporating both barriers and benefits can provide valuable field-tested guidance for increased programme planning for HIV testing.

## Introduction

The Joint United Nations Programme on HIV/AIDS (UNAIDS) estimates that more than 10 million people worldwide have contracted HIV since 2011.^1^ Despite public health efforts in key areas, such as West and Central Africa, declines in new HIV infection rates are ‘marginal’.^2^ To address stagnated HIV incidence, the World Health Organization (WHO), the United Kingdom’s Health Protection Agency and the United States’ Centers for Disease Control and Prevention (CDC) recommend expanded and integrated HIV testing strategies. This includes testing for all healthcare clients, including those utilising emergency services.^3,4,5^ The inclusion of the emergency department (ED) represents a viable and effective strategy for HIV testing in both the high-resource and resource-limited settings. Emergency departments provide unrestricted access to large numbers of patients who may not otherwise interact with the healthcare system, and have been shown to effectively identify new HIV infections in high-resource settings.^6,7,8^ Studies that have sought to quantify the effectiveness of ED-based HIV testing in low-resource settings are limited but have found an HIV prevalence of 2% – 43% with a proportion of 65% – 90% of previously undiagnosed HIV infection.^9^ In addition, EDs care for key high HIV-risk populations, such as drug and alcohol users, young men and sex workers who are missed in more traditional settings such as antenatal clinics.^1,10^

Despite the global HIV burden, national and international recommendations, as well as the demonstrated efficacy of ED-based testing in the United States, implementation has been inconsistent. A 2009 survey found that only 22% of EDs provided routine HIV testing, and of those, less than a third followed the recommended ‘opt-out’ format in the United States.^11^ A 2013 meta-analysis of HIV testing in the United Kingdom found that only 27.2% of eligible patients, as defined by the 2008 British HIV Association Guidelines, received HIV testing.^12^ Likewise, South African National Testing Guidelines call for universal HIV testing in all health facilities, including the ED, with pre- and post-test counselling; however, it is rarely implemented.^13^

Given this gap between practice and policy, a structured feasibility assessment may provide insight into barriers to implement effective and efficient ED-based HIV testing. Feasibility assessments are a key early component of dissemination and implementation of science and can have a large effect on an intervention’s acceptability, which in turn influences adoption, penetration and sustainability.^14^ The use of an appropriate and efficient assessment tool addresses the implementation component of a theoretical recommendation and provides prospective data on human and physical resources that need to be developed or supplemented to truly incorporate HIV testing into sustainable standards of practice. In addition, the use of a tool goes above and beyond more common feasibility assessments which do not proactively gather data but rather implement screening and then retrospectively evaluate their success using testing uptake as a proxy for feasibility.^15,16,17,18,19,20,21^ Assessment tools are available from the WHO, Family Health International, Partners in Health and other HIV/AIDS organisations.^22,23,24,25^ In this study, we seek to evaluate the usability and importance of a pre-implementation facility assessment tool in a low-resource setting where implementation of ED-based HIV testing is desperately needed.

### Methods

We performed a cross-sectional feasibility assessment of a convenience sample of four tertiary hospitals in the Eastern Cape of South Africa. Hospitals were selected in the Eastern Cape based on geographic accessibility, existing relationships with participating institutions and the prioritisation of research efforts by the Medical Research Council of South Africa. Institutional Review Boards at Johns Hopkins University and Walter Sisulu University approved the study (IRB number IRB00105801).

## Survey instrument

**FIGURE 1:**
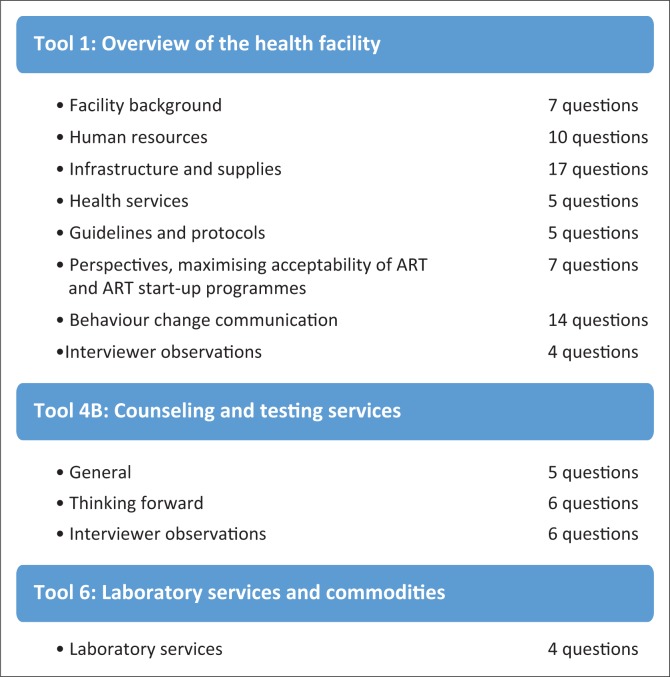
Tools summary.

We utilised three interview tools in accordance with guidelines from Family Health International’s Health Facility Tools to Assess Preparedness for HIV Service Delivery.^23^ The Guide consists of 13 tools that were designed to rapidly and comprehensively gather data regarding the availability and quality of essential elements of HIV services, organise and analyse the data, and plan for programme implementation. The Guide has been field tested in Cambodia, Ethiopia, Nigeria, Kenya, Senegal and Zambia and was designed to be flexible for adaption to meet individual site needs.^23^ The interview tools in our study were selected by both international and local researchers (authors M.W., B.H. and P.M.) based on applicability to the South African ED setting. Two of the tools were administered without change from the original text. The third tool, an assessment of laboratory services, was modified to only include information that could not be gathered from review of the National Health Laboratory Service of South Africa Scope of Services.^26^ The interview tools included yes/no, closed- and open-ended questions, as well as interviewer observations. This specific feasibility assessment toolkit was chosen based on a review of available tools and its previous use in low-resource settings. An overview of each of the three tools can be seen in Figure 1. The three tools in their entirety can be found at http://www.aidsdatahub.org/sites/default/files/documents/Health_Facility_Tools_to_Assess_Preparedness_for_HIV_Services_Delivery_Including_Antiretroviral_Therapy.pdf.

## Data collection

Interviewees consented to using the provided script and delivered their responses orally or via email. In-person interviews were conducted in the interviewees’ private offices. All interviews were conducted in English by the Johns Hopkins University researcher (M.W.) with facilitation from the Walter Sisulu University research partner (P.M.).

## Data analysis

All answers were recorded on the paper survey tools and later entered into Excel for side-by-side analysis of each question for all four sites. The majority of questions were closed-ended and direct responses were recorded. For open-ended questions, the researcher listed each response verbatim, then combined and tallied repeated answers. Interviewees selected answers from a finite list regarding barriers and benefits to ED-based HIV testing as well as provided open-ended responses. These answers were then combined into one table. Narrative answers that commented on the same problem, but with slightly different language (e.g. ‘manpower’ and ‘staff’), were grouped.

### Results

A total of 10 interviews were completed across four sites. Information was gathered from four lab supervisors, three physicians and three nurses. Interviewees are identified in Table 1.

**TABLE 1:** Interviewee role identification.

Facility	Interviewee	
	Too1 1 and Tool 4b	Tool 6
FH	Area manager (professional nurse)	Lab manager
NMH	Head of Department (physician) and nurse in charge (professional nurse)	Chemical pathologist
LH	Head of Department (physician) and area manager (professional nurse)	Lab manager
CMH	Head of Infectious Disease (physician)	Lab manager

LH, Livingstone Hospital; CMH, Cecilia Makiwane Hospital; FH, Frere Hospital; NMH, Nelson Mandela Hospital.

## Facility characteristics

All facilities are state-run tertiary academic hospitals and operate under Walter Sisulu University. All sites have 24-hour access to electricity (with back-up generators), water and computers. None of the EDs have designated laboratory personnel to perform point-of-care testing, and only one site, LH, has an in-department, part-time pharmacist. In-depth facility descriptions are shown in Table 2.

**TABLE 2:** Facility descriptions.

Facility	Type	Location	ED patients per day	Human resource availability†
FH	Urban	East London	140–150	Medical doctors: 11 Nursing staff:‡ 45 HIV tester and counsellors: 0
NMH	Rural	Mthatha	30–50	Medical doctors: 11 Nursing staff:‡ 24 HIV tester and counsellors: 0
LH	Urban	Port Elizabeth	260–330	Medical doctors: 14 Nursing staff:‡ 84 HIV tester and counsellors: 0
CMH	Rural	Mdantsane	80–100	Medical doctors: 10 Nursing staff:‡ 34 HIV tester and counsellors: 0

LH, Livingstone Hospital; CMH, Cecilia Makiwane Hospital; FH, Frere Hospital; NMH, Nelson Mandela Hospital.

† , Number of full-time, dedicated staff.

‡ , Includes professional nurses, staff nurses, enrolled nurses and nursing assistants.

ED, emergency department.

## Health services

Each of the four hospitals has inpatient and outpatient departments. In addition, all of the EDs provide physician-initiated, targeted HIV testing. Patients found to be HIV-positive are referred to their community antiretroviral (ARV) clinic using a standard referral form with the exception of Livingstone Hospital, which performs direct doctor-to-doctor sign-out on referred patents. Available health services at all hospitals include rapid and diagnostic HIV testing, ARV clinic, social services, pharmacy and post-exposure prophylaxis for employees only. Frere Hospital, Nelson Mandela Hospital and LH have on-site National Health Laboratories and are able to perform all HIV-related testing. Cecilia Makiwane Hospital sends specimens offsite for testing.

## Benefits and barriers to emergency department-based HIV testing

In addition to concrete information regarding provision of services related to HIV in the ED, we gathered subjective information from key informants regarding benefits and barriers to HIV testing in the ED. Responses to both open and closed-ended questions regarding testing barriers and potential benefits are included in Table 3. Each response is followed by the frequency it was listed in parentheses.

**TABLE 3:** Opportunities and barriers to emergency department–based HIV testing.

Benefits	Barriers
- Improved coordination with HIV referral system - Availability of reinforced ARV adherence counselling - Sharing of knowledge with outpatient clinics	- Staff shortages (4) - Physical space (2) - Perception that the ED is not the place for HIV testing as there are other dedicated HIV testing resources (duplication of services) (3) - Lack of time - Concerns regarding LTC (2) - Funding (2) - Insufficient staff education (2) - Health policy that HIV testing should be referred to clinics - Hospital policy does not allow for medication prescriptions for more than 7 days making it difficult to start ARVs

ARV, antiretroviral; ED, emergency department, LTC, linkage to care.

## Discussion

The use of a structured feasibility assessment tool demonstrated concrete barriers to ED-based HIV testing that will need to be addressed at the institutional level prior to implementing an ED-based HIV testing strategy. However, some of the identified barriers are likely to spark controversy and will require strategies for improvement beyond the ED. Use of a structured tool informed understanding of staffing shortages (mix as well as amount), the structure of ED services in the context of the institution and cultural barriers. The descriptive nature of these interview tools brings new illustrative information to the study of HIV testing implementation.^27^

This assessment clearly demonstrated the lack of available human resources to take on new responsibilities and projects, such as implementation of ED-based HIV testing. The number of full-time ED nurses ranged from 24 to 84 to serve large daily volumes of patients. Emergency department physician staffing only ranged from 11 to 14 full-time providers to serve the same population. The shortage of nurses and doctors in South Africa, specifically those with emergency training, is an ongoing problem and will need to be factored into any new programme implementation.^8,28,29^ In addition, none of the facilities has ancillary staff that could potentially take on the new responsibility of HIV testing and counselling, such as social workers, laboratory technicians or other cadres. These numbers demonstrate a clear barrier to ED-based HIV testing and help to create a more robust picture of the human resource constraints to successful implementation of the HIV testing policy. The question of availability and willingness of staff is cited in many accounts of ED-based HIV testing in both high- and low-resource settings.^20,30,31,32^

The feasibility assessment tool provided a structured approach to garner information at each of the facilities and how the ED functions as a part of the whole. For all facilities, HIV testing has been systematically and purposefully shifted to outpatient and community ARV clinics.^13^ This system of assigning a task to the most basic facility that can fulfil it has created both cultural and structural barriers to ED-based HIV testing, as well as missed opportunities to test new patients. None of the four EDs provided universal HIV testing, and none currently has the resources to initiate ARVs. This is problematic in the current HIV environment that calls for non-targeted testing and treatment regardless of symptoms, viral load or CD4+ count.^33^ In addition, the processing time for laboratory tests may not be conducive to the fast pace of the ED environment.

Finally, the structured interviews recorded perceived benefits and barriers to HIV testing. This information reinforced and illuminated much of the information gathered through the objective interview tools. Gathering these data describes institutional knowledge and individual attitudes. Items such as health and hospital policy need to be addressed in order to successfully introduce a change in practice and culture. In addition, the perception that the ED is not the venue for testing for communicable diseases appears to be ingrained in hospital culture, and a campaign to challenge this assumption may need to be incorporated into project planning. The prevalent themes of lack of provider knowledge, costs, insufficient time, insufficient space and linkage to care (LTC) issues are apparent in this setting and echo concerns noted in ED-barrier studies completed in the United States and the United Kingdom.^27,31,32,34,35,36^ The overall concept of reframing the role of the ED in public health initiatives is a prevalent theme in both high- and low-resource settings.^20,31,35^ In addition, Mumma and Suffaletto^30^ highlight the need for public health officials to recognise the many competing priorities of the ED and create a collaborative project that meets both the acute care and public health needs of the population.^30^

Implementation of Family Health International’s interview guides was simple, and the language was accessible to the interviewees, including those for whom English was not a first language. Some of the questions required both high-level institutional knowledge and department-level statistics (e.g. number of full-time and part-time employed nurses) that was difficult for some respondents to provide. The questionnaires are also lengthy which makes sustained quality data collection difficult, given that it requires interviews with key informants in busy leadership positions. In addition, the key question of funding a new HIV testing initiative is not addressed in this assessment. Our study did not utilise Family Health International’s provider or client attitudes tools but rather incorporated a more in-depth attitudes interview that included healthcare provider stigma and patient HIV knowledge at FH that has been previously described in this journal.^37^ Future pre-implementation efforts will need to continue to gather information on acceptability at all sites as it is a large component of cultural feasibility.

The use of this feasibility assessment provided essential information at the hospital level that, if expanded, could be used to influence policy at the institutional, provincial, state and even national level. In addition, given the documentation of the shortages of physical and human resources as well as staff buy-in found in this, and other feasibility assessments, it may be unethical to mandate new HIV testing strategies without accompanying solutions.^31,35^

In order to further inform the feasibility of ED-based HIV testing in these facilities, it will be important to administer both patient and provider questionnaires at all sites to evaluate cultural barriers, including stigma.^37^ In addition, the cost-effectiveness and financial feasibility of implementing a novel HIV testing programme will need to be studied. Given the time-prohibitive length of the surveys, it may also be beneficial to administer the tools in a setting that already provides ED-based HIV testing to determine which questions truly add value. This abbreviated questionnaire could then be validated. A priority matrix may also be a helpful prioritisation tool to organise results.

As this study was performed with a small convenience sample of four hospitals, it is limited in its generalisability to other institutions. The range of hospital resources and capacity is widely varied not only throughout South Africa but also across low-resource settings. In addition, interviewees provided information from their knowledge base and catchment areas; ED volumes, staffing numbers and laboratory times are estimates. Finally, bias is inherent to qualitative interviewing techniques. Structured interview guides were used to minimise interviewer level variation, but it cannot be completely eliminated.^38^

## Conclusion

This feasibility assessment helped to inform the possibility of initiating ED-based HIV testing by establishing an anticipatory list of barriers that will need to be addressed prior to initiation of a new testing strategy. Although potentially high yield, ED-based HIV testing in South Africa will need to have dedicated resources to shift the cultural perception of acute care versus public health mandates, as well as physical limitations such as funding, staff and space. Possible solutions to these obstacles could be incorporation of more traditional strategies, such as clinic-based testing, diverting testing resources to the inpatient setting owing to the captive audience of patients, or provision of at-home or self-testing kits to all ED patients.^39,40^ Taking advantage of blood samples already sent to the laboratory for add-on testing may also increase HIV testing volumes while minimising performing point-of-care testing on frontline staff.^41,42^
